# Multiple Triplet Metal‐Centered Jahn‐Teller Isomers Determine Temperature‐Dependent Luminescence Lifetimes in [Ru(bpy)_3_]^2+^

**DOI:** 10.1002/anie.202308803

**Published:** 2023-09-15

**Authors:** David Hernández‐Castillo, Roland E. P. Nau, Marie‐Ann Schmid, Stefanie Tschierlei, Sven Rau, Leticia González

**Affiliations:** ^1^ Institute of Theoretical Chemistry Faculty of Chemistry University of Vienna Währinger Str. 17 1090 Vienna Austria; ^2^ Doctoral School in Chemistry (DoSChem) University of Vienna Währinger Straße 42 1090 Vienna Austria; ^3^ Institute of Inorganic Chemistry I Ulm University Albert-Einstein-Allee 11 89081 Ulm Germany; ^4^ Technische Universität Braunschweig Department of Energy Conversion, Institute of Physical and Theoretical Chemistry Rebenring 31 38106 Braunschweig Germany; ^5^ Vienna Research Platform Accelerating Photoreaction Discovery University of Vienna Währinger Straße 17 1090 Vienna Austria

**Keywords:** Coordination chemistry, Ruthenium compounds, Photophysics, Temperature-dependent photoluminescence, Emission lifetimes

## Abstract

Understanding the factors that determine the luminescence lifetime of transition metal compounds is key for applications in photocatalysis and photodynamic therapy. Here we show that for ${\left[\mathrm{Ru}{\left(\mathrm{bpy}\right)}_{3}\right]}^{2+}$ (bpy = 2,2’‐bipyridine), the generally accepted idea that emission lifetimes can be controlled optimizing the energy barrier from the emissive triplet metal‐to‐ligand charge‐transfer (^3^MLCT) state to the thermally‐activated triplet metal‐centered (^3^MC) state or the energy gap between both states is a misconception. Further, we demonstrate that considering a single relaxation pathway determined from the minimum that is lowest in energy leads to wrong temperature‐dependent emission lifetimes predictions. Instead, we obtain excellent agreement with experimental temperature‐dependent lifetimes when an extended kinetic model that includes all the pathways related to multiple Jahn–Teller isomers and their effective reaction barriers is employed. These concepts are essential to correctly design other luminescent transition metal complexes with tailored emission lifetimes based on theoretical predictions.

## Graphical abstract

Time‐dependent emission lifetimes of [Ru(bpy)_3_]^2+^ are in excellent agreement with experimental values only when multiple pathways involving Jahn–Teller isomers of the ^3^MC excited states are considered.

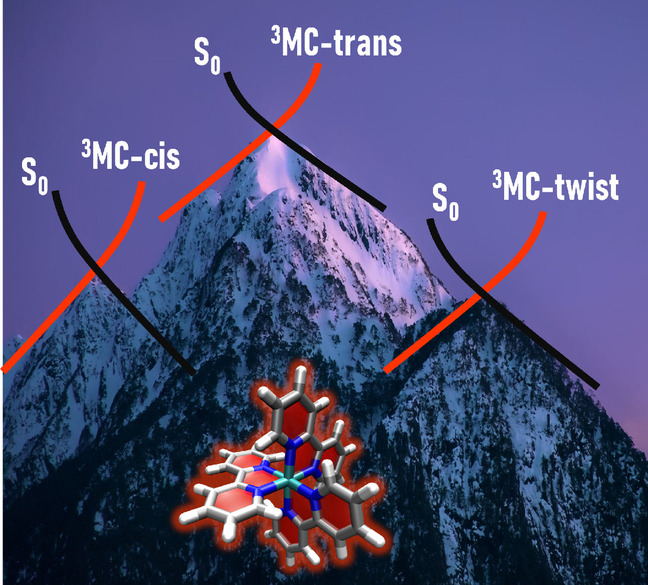

## Introduction

Ruthenium (Ru) polypyridine complexes displaying long luminiscence lifetimes are essential in many research areas, ranging from renewable energy including $H_{2}$ evolution,[^1^, ^2^] CO_2_ reduction,[^3^, ^4^] and dye‐sensitized solar cells,^[5]^ to photoredox synthetic organic catalysis^[6]^ and photodynamic therapy.^[7]^ The lifetime of the luminescent state is determined by competing deactivation reactions from the lowest triplet electronic state, which for such complexes is of metal‐to‐ligand charge‐transfer (^3^MLCT) character, i.e. a state where an electron nominally located at the Ru center is transferred to an orbital associated to the bipyridyl ligand.

**Figure 1:**
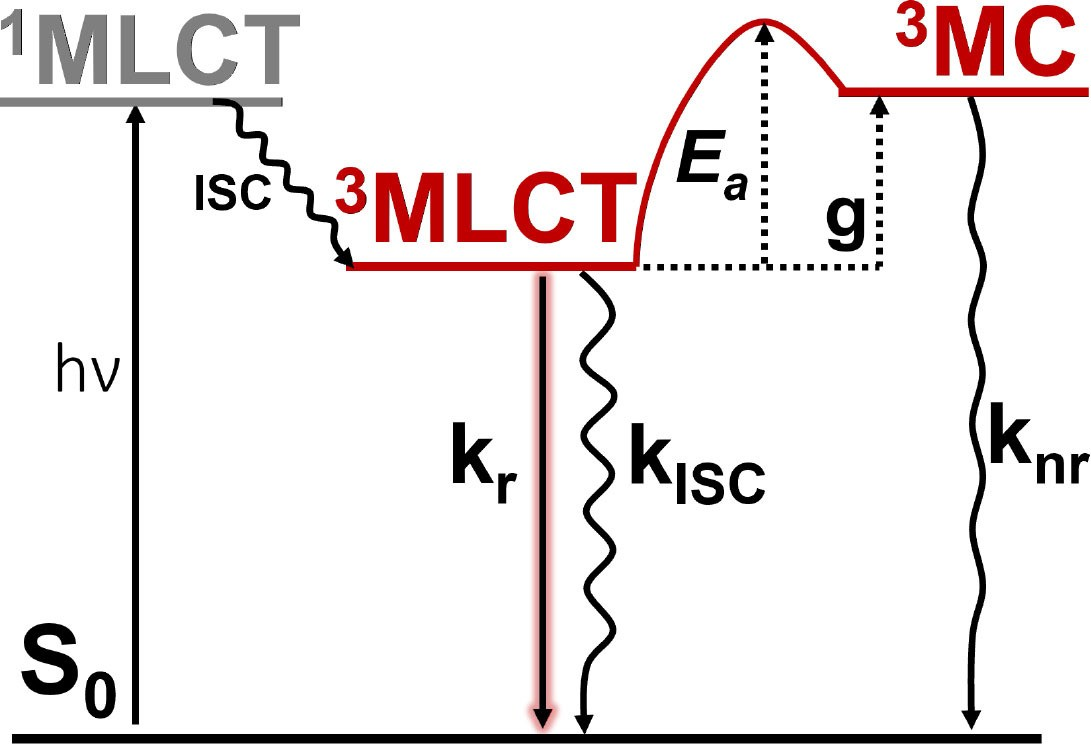
Simplified Jablonski diagram for Ru polypyridyl complexes. Upon irradiation, singlet metal‐to‐ligand‐charge‐transfer (^1^MLCT) states are populated, which via intersystem crossing (ISC) relax into the radiative ($k_{r}$ ) ^3^MLCT states. If sufficient thermal energy is available to overcome the barrier *E*_a_, relaxation from the lowest ^3^MLCT state to triplet metal‐centered (^3^MC) states can take place, from where the system deactivates to the electronic ground state non‐radiatively ($k_{\mathrm{nr}}$ ). g is defined as the energy gap between the lowest ^3^MLCT and ^3^MC states.

There exist examples where triplet lifetimes of Ru‐based compounds have been pushed up to microseconds.^[8]^ However, and despite encouraging experimental results employing extended bipyridine based π‐orbitals that lead to prolongation of lifetimes of luminescent states, it remains challenging to implement results from theory to design ligands with matching properties. There is a bottleneck that hinders leveraging synergies between theory and experiment, preventing the development of advanced photodynamic therapy agents for hypoxic environments^[9]^ and limiting applications that require small amounts of catalysts with long excited state lifetimes. An additional burden is that at room‐temperature – the ideal working condition for most applications – the lifetime and emission quantum yields of the ^3^MLCT states exponentially reduces.[^10^, ^11^, ^12^] This is because with increasing temperature, relaxation from the lowest ^3^MLCT state to thermally activated and non‐emissive metal‐centered triplet states (^3^MC), that ultimately lead to non‐radiative decay to the ground state, competes with emission (see Figure 1). On the other hand, recent results showed how raising the temperature up to 50 °C in light‐driven two‐electron reduction catalysis leads to 6.5 fold acceleration on catalytic performance,^[13]^ paving the way for obtaining photocatalysis faster than thermal catalysis. It is therefore of utmost importance to understand primary photochemical reaction steps and the factors that govern competing deactivation pathways in order to tailor emission lifetimes and ultimately optimize performance in photocatalysts.

The prototypical, tris(bipyridine)Ru complex, ${\left[\mathrm{Ru}{\left(\mathrm{bpy}\right)}_{3}\right]}^{2+}$ (bpy=2,2’‐bipyridine), is by far the most popular molecule when it comes to investigate temperature‐dependent phosphorescence lifetimes. Knowledge learnt from ${\left[\mathrm{Ru}{\left(\mathrm{bpy}\right)}_{3}\right]}^{2+}$ is often readily transferred to related Ru derivatives^[14]^ and other luminescent transition metal complexes,[^15^, ^16^] even if there is no evidence that the behavior of ${\left[\mathrm{Ru}{\left(\mathrm{bpy}\right)}_{3}\right]}^{2+}$ will be general. Experimental insight on the deactivation from the lowest ^3^MLCT state is commonly attained by performing variable temperature photoluminescence measurements. The obtained emission lifetimes are then fitted against temperature by using Eq. 1.

(1) $$\tau_{\mathrm{emission}}=\frac{1}{k_{r}+k_{\mathrm{ISC}}+k_{\mathrm{nr}}}$$

In this Equation (see also Figure 1), $k_{r}$ is the radiative rate constant of the emissive ^3^MLCT state, $k_{\mathrm{ISC}}$ the intersystem crossing (ISC) rate from the emissive state to the ground state $S_{0}$ and $k_{\mathrm{nr}}$ is the thermally‐activated non‐radiative rate, which can be expressed by the Arrhenius‐like rate constant,

(2) $$k_{\mathrm{nr}}={\mathrm{Ae}}^{\frac{-\Delta E}{\mathrm{RT}}}$$

Note that the rate constants k_r_ and k_nr_ are both decay processes starting from the same electronic excited state (^3^MLCT) but through different pathways. The term $k_{\mathrm{nr}}$ allows to correlate emission lifetimes with the deactivation through the non‐emissive ^3^MC states. The landmark works from Van Houten and Watts^[10]^ followed up by Caspar and Meyer^[17]^ provided some of the earliest experimental values for the ΔE by applying Equation 2 to a wide range of temperatures and solvents. Following these pioneering papers, ΔE has been ever since interpreted as either the energy gap (*g*) between the lowest ^3^MLCT and ^3^MC states[^10^, ^18^, ^19^, ^20^, ^21^] or the energy barrier (*E*_a_) between both states[^14^, ^17^, ^22^, ^23^, ^24^, ^25^, ^26^, ^27^, ^28^, ^29^, ^30^] (see Figure 1).

In this paper, we show that this interpretation (associated with the simple Jablonski diagram of Figure 1) is an oversimplification that neglects other temperature‐dependent non‐radiative pathways and the role of the crossing points among relevant states. We claim that, the widespread assumption that ΔE is only related to a single relaxation process is in general an ambiguous definition and leads to wrong theoretical predictions of the emission lifetimes. To date, such wrong predictions are erroneously attributed to the inaccuracy of the employed levels of theory, as very small changes in the ΔE term of Eq. 2 can easily return reaction rates with uncertainties that extend several orders of magnitude. Problematic consequences of this misconception are that it can lead to misleading fortuitous linear correlations between the activation energy *E*_a_ and the experimentally measured quantum efficiency, to false theoretical benchmarks, and to wrong structure–property relationships where compounds with increasing ^3^MLCT‐^3^MC energy gaps are optimized but emission lifetimes do not necessarily increase.

As we will show here, these mismatches are linked to the use of the wrong kinetic model for the non‐radiative decay of ${\left[\mathrm{Ru}{\left(\mathrm{bpy}\right)}_{3}\right]}^{2+}$ and the use of the concept of *rate‐determining step* instead of *rate‐determining state(s)*.[^31^, ^32^, ^33^, ^34^] We show here that the deactivation of ${\left[\mathrm{Ru}{\left(\mathrm{bpy}\right)}_{3}\right]}^{2+}$ is a process more complex than previously assumed from the experimental standpoint.[^10^, ^17^] Based on comprehensive and accurate quantum chemical calculations of the complete energetic profile from the emissive ^3^MLCT state, it is possible to calculate emission lifetimes, if the correct kinetic model is used. The validity of our approach on ${\left[\mathrm{Ru}{\left(\mathrm{bpy}\right)}_{3}\right]}^{2+}$ is corroborated by temperature‐dependent emission lifetimes measurements.

## Results and Discussion

**Figure 2:**
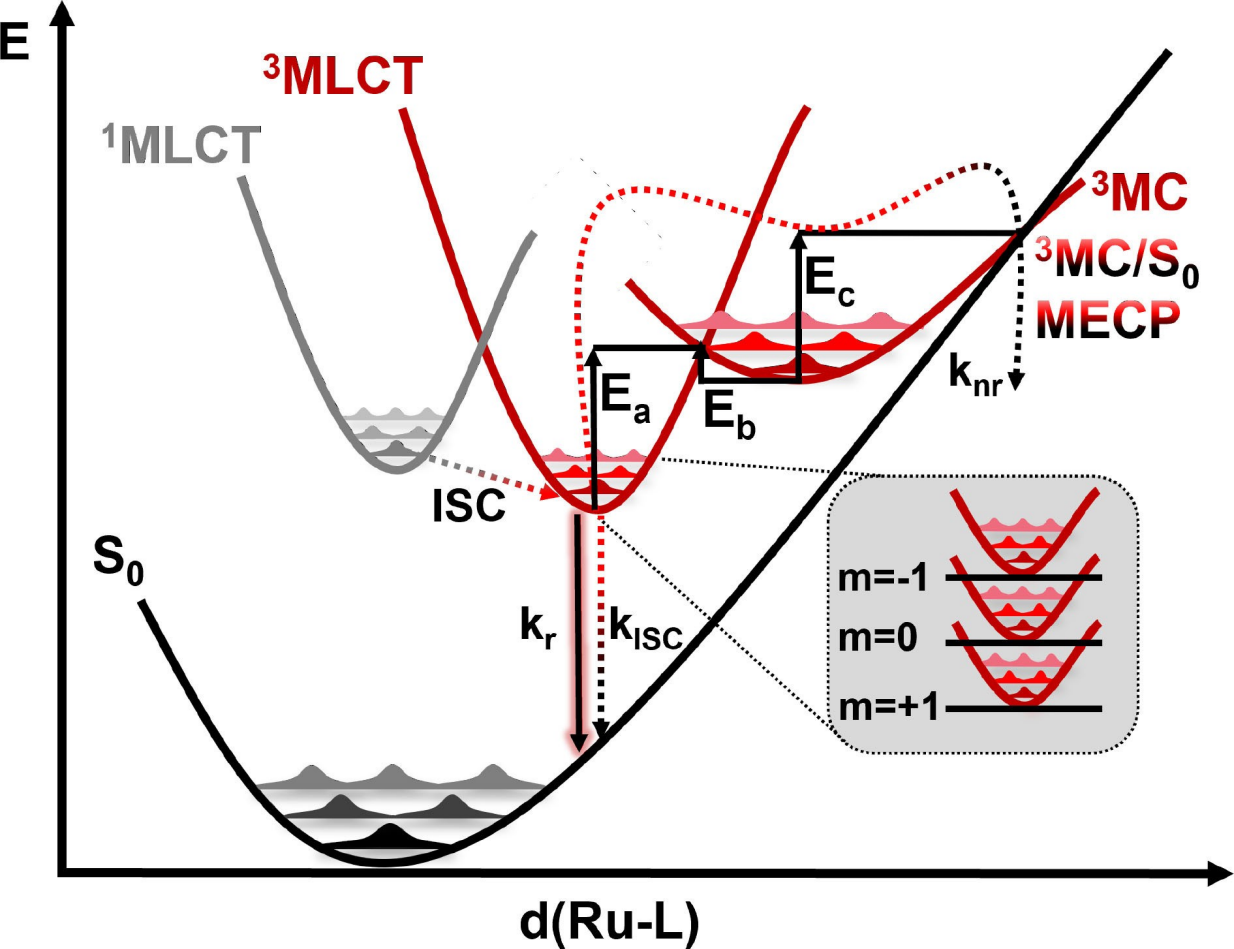
Potential energy surfaces depicting essential photophysical process for the deactivation of ${\left[\mathrm{Ru}{\left(\mathrm{bpy}\right)}_{3}\right]}^{2+}$ and likely related Ru‐based complexes, see text. Note that k_nr_ denotes the overall non‐radiative rate starting at the ^3^MLCT state to the ground state S_0_, through the ^3^MC state.

Figure 2 shows a more complex scheme of the key photophysical processes associated to the deactivation of ${\left[\mathrm{Ru}{\left(\mathrm{bpy}\right)}_{3}\right]}^{2+}$ and often also attributed to other Ru‐metal complexes. Upon light irradiation, a manifold of singlet ^1^MLCT states is populated. In ${\left[\mathrm{Ru}{\left(\mathrm{bpy}\right)}_{3}\right]}^{2+}$ , ISC to the triplet ^3^MLCT manifold ensues in less than 30 fs,[^35^, ^36^, ^37^] followed by internal conversion to the lowest ^3^MLCT state, from where phosphorescence takes place with a rate constant $k_{r}$ . Spin–orbit coupling lifts the three‐fold degeneracy of the triplet states, making a Boltzmann statistical treatment of the lowest three‐spin sublevels mandatory for an accurate calculation of $k_{r}$ . From the ^3^MLCT state, non‐radiative decay via ISC to the electronic ground state S_0_ is also possible. This process is temperature‐dependent through the increase of the population of high‐energy vibrational states and it is associated with the rate constant $k_{\mathrm{ISC}}$ . Upon increasing the temperature, ^3^MC states can also be populated from the 3MLCT state, leading to irreversible non‐radiative deactivation ($k_{\mathrm{nr}}$ ) to the S_0_ via the ^3^MC/S_0_ minimum energy crossing point (MECP). It is precisely the increasing population of the lowest ^3^MC states at room‐ or higher temperature what leads to a reduction in emission lifetimes.^[17]^

**Figure 3:**
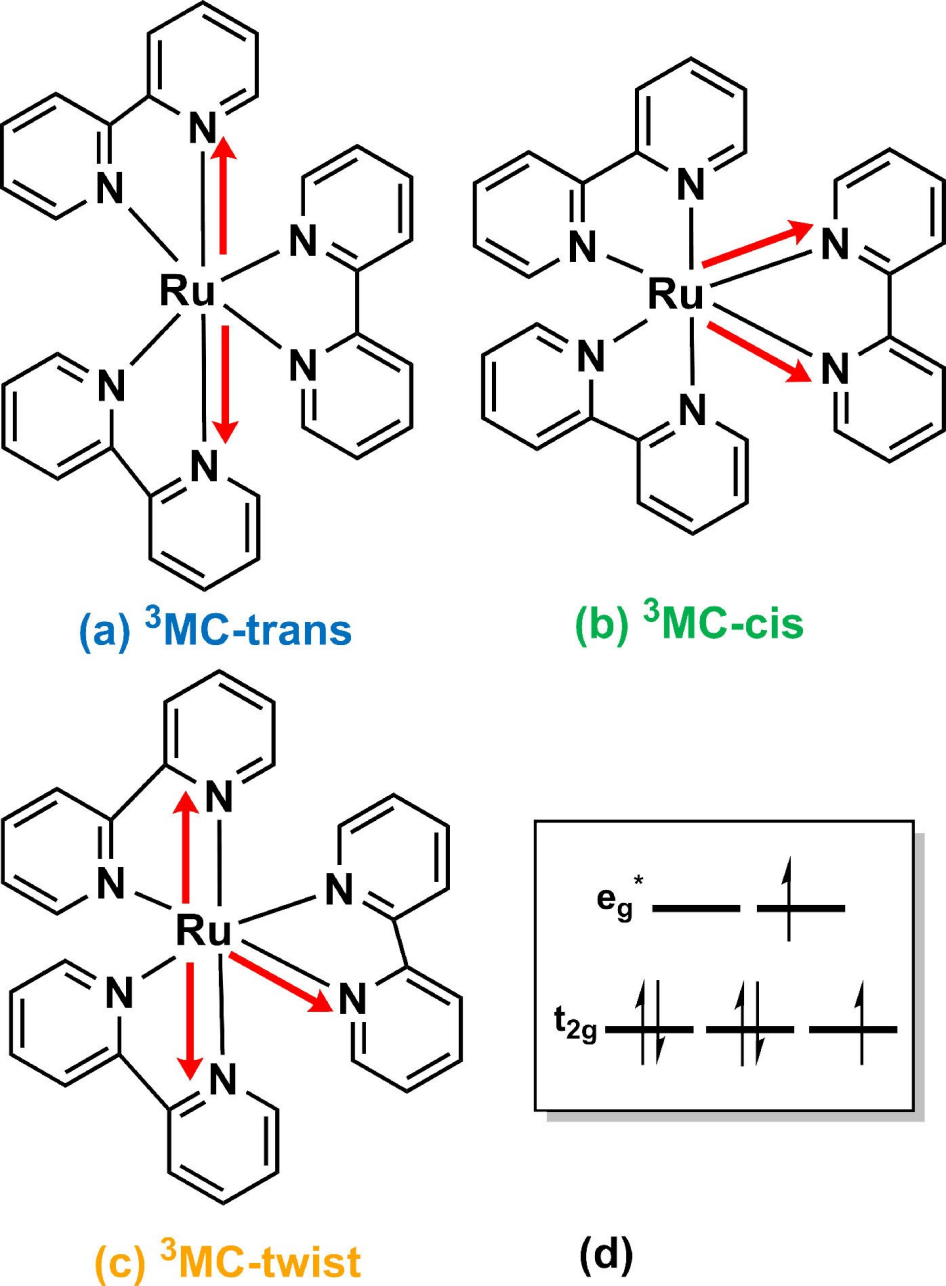
Trans (a), cis (b) and twist (c) Jahn–Teller isomers of ${\left[\mathrm{Ru}{\left(\mathrm{bpy}\right)}_{3}\right]}^{2+}$ with ^3^MC character, as described in earlier studies.[^27^, ^40^, ^42^, ^43^] Red arrows indicate the characteristic elongated Ru−N bonds, steaming from the population of σ ‐antibonding $e_{g}^{*}$ orbitals (d).

Due to its short‐lived nature, the experimental structural characterization of the ^3^MC states is challenging.[^25^, ^38^, ^39^] Likely, the first proposal of a ^3^MC geometry in ${\left[\mathrm{Ru}{\left(\mathrm{bpy}\right)}_{3}\right]}^{2+}$ came from the theoretical work of Alary et al.^[40]^ They predicted a geometry with two elongated axial Ru−N bonds^[40]^ – a Jahn–Teller^[41]^ (JT) distorted structure with symmetry decreased from D_3_ to C_2_. This structure is labelled here the ^3^MC‐trans JT isomer (Figure 3a). The presence of small imaginary frequencies indicated that this was not a true minimum and although the calculations nonphysically placed this geometry energetically lower than the emissive ^3^MLCT state,^[40]^ this was considered for a long time the key structure to explain the non‐radiative decay of ${\left[\mathrm{Ru}{\left(\mathrm{bpy}\right)}_{3}\right]}^{2+}$ and many homoleptic 2,2’‐bipyridine analogs. A decade later, the structure was reoptimized with a better level of theory and the true ^3^MC‐trans minimum was identified.^[27]^ In line with experimental expectations, its energy lied above that of the ^3^MLCT state.[^10^, ^17^, ^38^] Interestingly, the same authors had also reported another two minima of ^3^MC character. One of them^[42]^ is characterized by two elongated Ru−N bonds on the same bipyridyl ligand – a structure here denoted as the ^3^MC‐cis JT isomer (Figure 3b). The other minimum^[43]^ features two Ru−N bond elongations in two different bipyridyl ligands and a N−Ru−N angle (of the respective elongated Ru−N bonds) close to 90 degrees – thus, we label it the ^3^MC‐twist JT isomer (Figure 3c). The latter two geometries have been discussed in the literature only in the context of the photorelease of the bipyridine ligand(s)[^42^, ^43^] but never to calculate luminescence lifetimes. Below we show how the fate of the ^3^MLCT state strategically depends less on the ^3^MC‐trans minimum and more on the ‐cis and ‐twist JT isomers –hitherto neglected.

**Figure 4:**
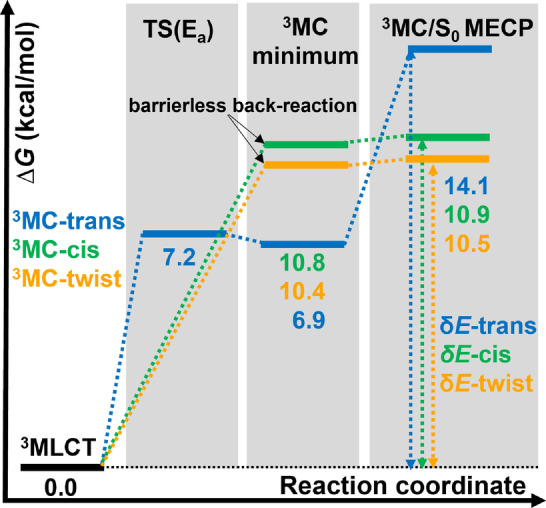
Computed relative Gibbs free energies at 298.15 K for the non‐radiative decay of the lowest ^3^MLCT state through the ^3^MC‐trans (blue), ^3^MC‐cis (green) and ^3^MC‐twist (orange) JT isomers. *δE* is the energy span or effective reaction barrier for each respective pathway (see discussion in the text). Energies are given in kcal/mol relative to the ^3^MLCT state (0.0 kcal/mol). Level of theory: B2GP‐PLYP−D3/def2‐QZVPP@CPCM(acetonitrile)//B3LYP−D3/def2‐SVP@CPCM(acetonitrile).

First, we set to calculate the relative free energies associated to the decay of ${\left[\mathrm{Ru}{\left(\mathrm{bpy}\right)}_{3}\right]}^{2+}$ considering the ^3^MC‐trans (blue in Figure 4), ^3^MC‐cis (green) and ^3^MC‐twist (orange) isomers. As we aim at predictions as accurate as possible, we use a double hybrid functional[^44^, ^45^, ^46^] and extensive basis sets (see Section S1 of Supporting Information for further computational details). This level of theory achieves accuracy beyond the one obtained by the popular B3LYP hybrid functional, employed in previous works.[^27^, ^28^, ^42^]

For the trans JT isomer, we found a transition state between the lowest ^3^MLCT and ^3^MC state here predicted at 7.2 kcal/mol, as suggested in previous computational studies.[^27^, ^42^] For the ‐cis and ‐twist structures, never investigated in the past in this context, we found no transition state, indicating that the latter pathways are barrierless (Figure 4). The corresponding ^3^MC minima are predicted at 6.9, 10.8 and 10.4 kcal/mol for the trans‐, cis‐ and twist‐JT isomers, respectively. With this information at hand, one could be tempted to conclude that the pathway through the ^3^MC‐trans isomer is the only one relevant for the emission of ${\left[\mathrm{Ru}{\left(\mathrm{bpy}\right)}_{3}\right]}^{2+}$ since it has the lowest ^3^MLCT→ ^3^MC reaction barrier (7.2 kcal/mol) in comparison to the cis‐isomer (10.8 kcal/mol) and the twist‐isomer (10.4 kcal/mol). However, keeping this line of reasoning, the computed reaction barrier to reach the ^3^MC‐trans minimum (7.2 kcal/mol) or the energy gap between both states (6.9 kcal/mol) would have to match the experimental ΔE value of 10.9 kcal/mol ($3800\;{\mathrm{cm}}^{-1}$ ) obtained by Caspar and Meyer in their luminescent measurements^[17]^ but they do not. Related mismatches have been justified in the past by the inherent errors of the employed level of theory. Here we disregard this argument. First, because the employed level of theory and second, and most importantly, because there is no experimental evidence that the derived value of 10.9 kcal/mol should be really ascribed to the *E*_a_ activation barrier or the energy gap *g* in ${\left[\mathrm{Ru}{\left(\mathrm{bpy}\right)}_{3}\right]}^{2+}$ (recall Figure 1).

To challenge this interpretation, we consider the following reaction in steady‐state regime,
(3)

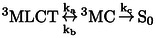




which describes all the steps starting from the ^3^MLCT state through a single ^3^MC state. Each involved rate can be calculated going from an energy‐representation to a k‐representation using the Eyring's equation,

(4) $$k=\kappa \frac{k_{B}T}{h}e^{\frac{-\Delta G^{\neq }}{\mathrm{RT}}}$$

where $\Delta G^{\neq }$ is the Gibbs free energy of activation, $k_{B}$ is the Boltzmann constant, h is Planck's constant and *κ* is the transmission coefficient set to 1 within the classical formulation of transition state theory.^[47]^ For the chemical reaction formulated in equation 3, the total reaction rate $k_{\mathrm{nr}}$ can be then formulated as

(5) $$k_{\mathrm{nr}}=\frac{k_{c}*k_{a}}{k_{b}+k_{c}}.$$

If the deactivation via the ^3^MC state through a 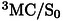 MECP would be much faster than the back reaction of the ^3^MC state to the ^3^MLCT state, i.e. $k_{c}\gg k_{b}$ , then $k_{\mathrm{nr}}=k_{a}$ and the ^3^MLCT/^3^MC‐trans reaction barrier would dominate the decay of the emissive ^3^MLCT state. This scenario is often presumed in the literature for ${\left[\mathrm{Ru}{\left(\mathrm{bpy}\right)}_{3}\right]}^{2+}$ , implying that the 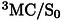 MECP should be energetically below the transition state of the ^3^MLCT→ ^3^MC reaction step. However, this is an ad‐hoc assumption unsupported by the reaction profile computed by us (Figure 4) and others.^[28]^ Instead, our calculations will evidence that $k_{a}$ alone and from a single process, cannot determine the overall rate of the non‐radiative decay through the ^3^MC state.

Since the ^3^MLCT/^3^MC step (Equation 3) has the smallest rate constant ($k_{a}$ ), it is not surprising that the ^3^MC states have been so far the target to derive structure/property relationships in the hope that they provide design rules for long‐lived luminescent ^3^MLCT states. Furthermore, and perhaps for historical reasons, only ^3^MC states with a trans distortion have been deployed in this endeavor. However, as highlighted in several publications,[^31^, ^32^, ^33^, ^34^] the concept of a *rate‐determining step* when rationalizing reaction mechanisms is often a deficient and ambiguous definition. Instead, the overall rate control of a given process is governed by intermediates and transition states that are referred as the *rate‐determining states* – these are the states providing the highest effective energy barriers. In our case (Figure 4), the effective energy barrier should be given by the energetic difference between the ^3^MLCT minimum and the 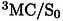 MECP – a quantity that can be defined for each of the three ^3^MC‐trans‐, cis‐ and twist‐ reaction pathways. This effective energy barrier is known as the *energetic span* (*δE*), which is why this conceptualization is known in the literature as the *energetic span model*.[^31^, ^32^, ^33^, ^34^]

Under this new light, the experimentally derived^[17]^ ΔE value of 10.9 kcal/mol (Equation 1) should correspond to the *energetic span* between the ^3^MLCT state and the 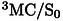 MECP. An easy test to identify such rate determining states is to simulate the degree of rate control[^48^, ^49^] of the intermediates and transition states/crossing points. This can be done by monitoring the changes in the overall reaction rate when doing differential changes in the relative energies of individual states involved in the mechanism (Section S2).

According to our calculations (Figure 4), the decay via the ^3^MC state involves *energetic spans δE* of 14.5 kcal/mol, 10.9 kcal/mol, and 10.5 kcal/mol for the trans‐, the ‐cis and the ‐twist JT isomers, respectively. Both, cis‐ and twist‐ effective energy barriers are, within the error of the method, in semi‐quantitative agreement with the measured ΔE ^[17]^ while the trans‐energy is off. Importantly, we can already conclude that, (i) ΔE should neither be associated to the energy barrier *E*_a_ from the ^3^MLCT to a ^3^MC state nor to the energy gap *g* between both states, but instead to the *energetic span* and (ii) the typical ^3^MC‐trans decay pathway has no weight determining the fate of the emissive ^3^MLCT state, but instead both the ^3^MC‐cis and ‐twist reaction pathways must contribute to the decay of ${\left[\mathrm{Ru}{\left(\mathrm{bpy}\right)}_{3}\right]}^{2+}$ and thus to the lifetime of the emissive ^3^MLCT state. At this point, it is fair to note that Escudero^[50]^ proposed to quantify the term ΔE in Equation 2 as $E_{\lim}$ which, as mathematically defined in his paper, should have corresponded to the *energetic span δE*; however, he defined it as “the activation energy for the limiting step”, what has led to misuses.^[51]^

With our new protocol at hand, we now aim at connecting the energy profile obtained from the quantum chemical calculations with measured temperature‐dependent emission lifetimes. Calculations of temperature‐dependent photoluminescent properties have remained elusive with rare exceptions, such as the work of Escudero and co‐workers on three Ir(III) complexes^[52]^ that share similarities in their luminescence decay with ${\left[\mathrm{Ru}{\left(\mathrm{bpy}\right)}_{3}\right]}^{2+}$ . The authors were able to compute all the terms of Equation 1 but obtained only qualitative agreement with experimental data as the emission quantum yields were underestimated in all cases by factors ranging between 1.35 and 11.^[52]^

Early experimental emission lifetimes of ${\left[\mathrm{Ru}{\left(\mathrm{bpy}\right)}_{3}\right]}^{2+}$ , from which the ΔE value of 10.9 kcal/mol was inferred, were recorded in acetonitrile from 77 to 300 K.^[17]^ In this low temperature regime, it is well‐known^[52]^ that the temperature dependency of $k_{\mathrm{ISC}}$ and $k_{r}$ dominates, while at higher temperatures $k_{\mathrm{nr}}$ dominates. This means that, the higher the temperature, the more accurate will be the experimental evaluation of ΔE via Equation 1. This prompted us to revisit the experimental data for ${\left[\mathrm{Ru}{\left(\mathrm{bpy}\right)}_{3}\right]}^{2+}$ in acetonitrile in the range from 280 K–350 K (see experimental details in section S3).

**Figure 5:**
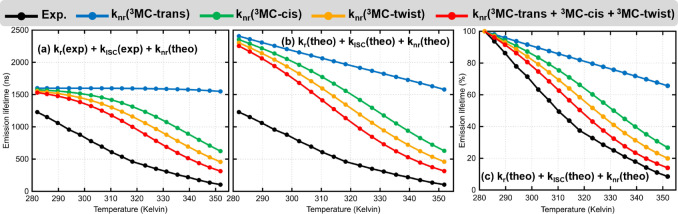
Temperature‐dependent emission lifetimes of ${\left[\mathrm{Ru}{\left(\mathrm{bpy}\right)}_{3}\right]}^{2+}$ in ns (a and b) and in percentage (c). Experimental values are given by the black line. Blue, green and orange lines are the computational lifetimes obtained using the non‐radiative rates k_nr_ of the ^3^MC‐trans, ^3^MC‐cis or ^3^MC‐twist isomers, respectively. Red line considers all three pathways, as in equation 6. Panel (a) shows computational results obtained using experimental k_r_ and k_ISC_ values. Panel (b) and (c) use theoretically calculated k_r_ and k_ISC_ values.

The experimental temperature‐dependent emission lifetimes are plotted in Figure 5a (black line). These values shall be compared to the computational lifetimes obtained from Equation 1. For the rate constants $k_{r}$ and $k_{\mathrm{ISC}}$ , we first use values estimated experimentally (Section S3) so that the comparison between experiment and theory can focus on the computational accuracy of $k_{\mathrm{nr}}$ , dominant at high temperatures. For the $k_{\mathrm{nr}}$ , we employ Equation 5, where the rate constants $k_{a,b,c}$ are calculated from the Gibbs free activation energies $\Delta G_{a,b,c}^{\neq }$ (recall Figure 2). Using the quantum chemical values computed for the ^3^MC‐trans, ^3^MC‐cis or ^3^MC‐twist pathways (Section S2, Table S8) results in the blue, green and orange plots of Figure 5a.

The curves computed with the ^3^MC‐cis and ^3^MC‐twist JT isomers show very good agreement with the experiment. Moreover, the fact that the curves associated to both JT isomers are essentially the same, confirms that both pathways are competitive. The similar behavior of the two isomers and their different behavior when compared to the ^3^MC‐trans isomer can be rationalized from the character of the populated e_g_* orbitals at the ruthenium metal center. The ^3^MC‐trans isomer shows population of a $d_{z^{2}}$ ‐like dσ* orbital, while the ^3^MC‐cis and ^3^MC‐twist JT isomers show a populated $d_{x^{2}-y^{2}}$ ‐like dσ* orbital (see Figure S3).[^27^, ^42^, ^43^] The character of the populated antibonding orbitals, when going from the emissive ^3^MLCT to a metal centered excited state, is also consistent with the corresponding distorted Ru−N bond (Figure S3).

In contrast, the calculations clearly show that at higher temperatures the emission lifetimes are not governed by the pathway involving the ^3^MC‐trans JT isomer (blue line). This is in stark contrast to previous theoretical studies on ruthenium‐[^14^, ^53^, ^54^, ^55^, ^56^, ^57^, ^58^, ^59^] and iridium‐[^11^, ^50^, ^52^, ^60^, ^61^] complexes, which focus on one isomer, either the prototypical trans‐isomer or just the lowest‐lying MC state, in order to compare or explain experimental data.

From our results, we claim that the experimentally measured $k_{\mathrm{nr}}$ should be rather the result of considering all deactivation pathways on the same footing. Hence, we also calculate the emission lifetime as,6

(6) $$\tau_{\mathrm{emission}}^{'}=\frac{1}{k_{r}{+k}_{\mathrm{ISC}}{+k}_{\mathrm{nr}}{(}^{3}\mathrm{MC}-\mathrm{trans}){+k}_{\mathrm{nr}}{(}^{3}\mathrm{MC}-\mathrm{cis}){+k}_{\mathrm{nr}}{(}^{3}\mathrm{MC}-\mathrm{twist})}$$

where $k_{\mathrm{nr}}{(}^{3}\mathrm{MC}-\mathrm{trans})$ , $k_{\mathrm{nr}}{(}^{3}\mathrm{MC}-\mathrm{cis})$ and $k_{\mathrm{nr}}{(}^{3}\mathrm{MC}-\mathrm{twist})$ are the non‐radiative decay rates through the ^3^MC‐trans, ^3^MC‐cis and ^3^MC‐twist pathways, respectively. Gratifyingly, the obtained result (red curve in Figure 5a) further improves the agreement towards the experimental data, confirming the validity of the extended Equation 6. Remaining small deviations (for instance, the position of the concave‐to‐convex inflexion point) can be attributed to the level of theory and possibly to the fact that we are using a constant value for $k_{\mathrm{ISC}}$ and $k_{r}$ , estimated from the experimental measurements. We recall that, a deviation of 1 kcal/mol in the estimated barriers can lead to an order of magnitude deviation in the rates (equation 4), so probably we have reached the limit of the current electronic structure methods.

A potential improvement of our theoretical estimates of lifetimes could be obtained by including temperature‐dependent $k_{\mathrm{ISC}}$ and $k_{r}$ rates. Accordingly, we compute $k_{\mathrm{ISC}}$ and $k_{r}$ (Section S1) to evaluate the emission lifetime in a fully theoretical setup. The results, shown in Figure 5b (for the individual JT isomers and as well as the sum), are similar to those of Figure 5a, i.e., at higher temperatures the pathway via the trans JT isomer alone cannot reproduce the emission lifetimes; rather, the pathways via the cis and twist JT isomers are responsible of the decay. The agreement is considerably better at higher temperatures due to our better description of $k_{\mathrm{nr}}$ , which then becomes the dominant decay. The errors at low temperature are probably rooted on the harmonic approximation of the calculation of the $k_{\mathrm{ISC}}$ . For comparison, emission lifetimes predicted when using k_a_ (^3^MLCT→^3^MC) instead of k_nr_ (^3^MLCT→^3^MC→S_0_) through equation 6 are almost zero (Figure S5). This result reinforces the consequences associated with incorrectly formulating the non‐radiative decay rate of the ^3^MLCT excited state (see further discussion in section S2.4). Figure 5c shows the emission lifetimes as a percentage plot, taking the values from panel b. Again here, we note the excellent agreement with the experiment resulting from including all the JT isomers in the calculation of the non‐radiative constant. In contrast, the trans JT isomer alone cannot account for the temperature behavior, and it is worse as the temperature increases.

## Conclusion

**Figure 6:**
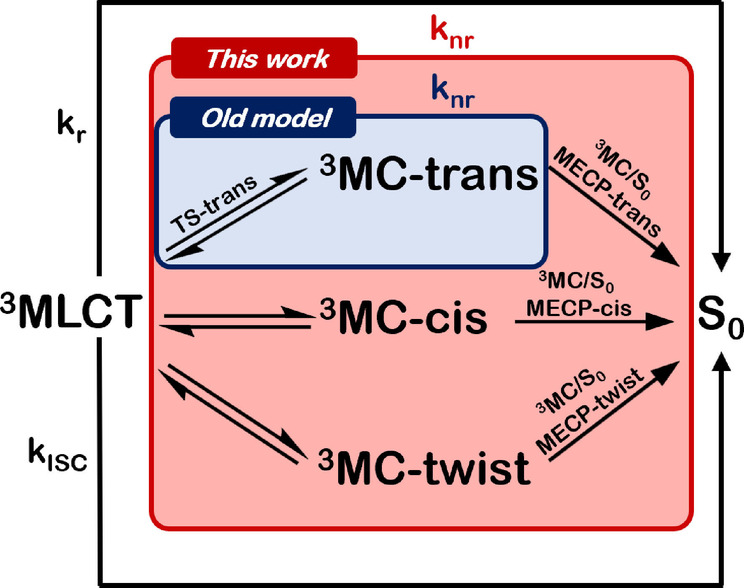
Conspectus of kinetic models proposed in the past and in this work for ${\left[\mathrm{Ru}{\left(\mathrm{bpy}\right)}_{3}\right]}^{2+}$ .

We demonstrate that the long‐standing viewpoint that emission lifetimes of ${\left[\mathrm{Ru}{\left(\mathrm{bpy}\right)}_{3}\right]}^{2+}$ and related compounds can be controlled tuning the energy barrier from the emissive ^3^MLCT to the thermally‐activated ^3^MC state or the energy gap between both states is in general not true. Furthermore, focusing on the *rate‐determining step*, and thus on a single relaxation pathway, is an oversimplification that leads to wrong predictions of lifetimes in ${\left[\mathrm{Ru}{\left(\mathrm{bpy}\right)}_{3}\right]}^{2+}$ . For this complex, this means that considering only the decay pathway related to the lowest ^3^MC minimum (the trans JT distorted isomer) returns emission lifetimes that significantly deviate from the experiment with increasing temperature as the non‐radiative reaction rate becomes dominant. Instead, we show here that it is possible to calculate quasi‐quantitative temperature‐dependent lifetimes when the pathways related to all the JT isomers are considered and the concept of *rate‐determining states* is employed. The two kinetic models are summarized in Figure 6, highlighting the difference in describing the non‐radiative rate through the metal‐centered excited states by either only the ^3^MC‐trans isomer (as in the literature so far) or via all different JT isomers (this work). Since the use of a single reaction pathway (i.e., a single ^3^MC geometry) and/or the lowest ^3^MC excited state to calculate luminescence lifetimes is very much rooted in the literature (rate‐limiting step), we hope that this work not only advises caution, but sparks reassessment of mechanistic interpretations of the photophysical/photochemical processes in transition metal complexes involving ^3^MC states.

Translating these notions from ${\left[\mathrm{Ru}{\left(\mathrm{bpy}\right)}_{3}\right]}^{2+}$ to other luminescent transition metal complexes is indispensable to guide the design of compounds with tailored emission lifetimes. Depending on the specific compound, the relative position of the ^3^MC minima and their deactivation towards the ^3^MLCT minima and the ground state can be different, affecting the weight between the different JT isomers

## Contributions

DHC: methodology, calculations, analysis, and writing original draft. REPN: synthesis, purification, characterisation and lifetime measurements. MAS: analysis of experimental data. ST: experimental design. SR: supervision of experimental work, editing the manuscript and funding acquisition. LG: conceptualization, analysis, supervision of theoretical work, review and editing the manuscript, project administration and funding acquisition. All authors have read and agree with the manuscript.

## Conflict of interest

No conflict of interest.

1

## Supporting information

As a service to our authors and readers, this journal provides supporting information supplied by the authors. Such materials are peer reviewed and may be re‐organized for online delivery, but are not copy‐edited or typeset. Technical support issues arising from supporting information (other than missing files) should be addressed to the authors.

Supporting Information

Supporting Information

## Data Availability

The data that support the findings of this study are available from the corresponding authors upon reasonable request.
